# Increased Autoimmunity in Individuals With Down Syndrome and Moyamoya Disease

**DOI:** 10.3389/fneur.2021.724969

**Published:** 2021-09-08

**Authors:** Jonathan D. Santoro, Sarah Lee, Anthony C. Wang, Eugenia Ho, Deepti Nagesh, Mellad Khoshnood, Runi Tanna, Ramon A. Durazo-Arvizu, Melanie A. Manning, Brian G. Skotko, Gary K. Steinberg, Michael S. Rafii

**Affiliations:** ^1^Division of Neurology, Department of Pediatrics, Children's Hospital Los Angeles, Los Angeles, CA, United States; ^2^Department of Neurology, Keck School of Medicine at the University of Southern California, Los Angeles, CA, United States; ^3^Department of Neurology, Stanford University School of Medicine, Palo Alto, CA, United States; ^4^Department of Neurosurgery, University of California, Los Angeles, Los Angeles, CA, United States; ^5^Biostatistics Core, Department of Pediatrics, Keck School of Medicine at the University of Southern California, Los Angeles, CA, United States; ^6^Department of Pediatrics, Stanford University School of Medicine, Palo Alto, CA, United States; ^7^Department of Pathology, Stanford University School of Medicine, Palo Alto, CA, United States; ^8^Department of Pediatrics, Harvard Medical School, Boston, MA, United States; ^9^Division of Medical Genetics and Metabolism, Department of Pediatrics, Massachusetts General Hospital, Boston, MA, United States; ^10^Department of Neurosurgery, Stanford University School of Medicine, Palo Alto, CA, United States; ^11^Alzheimer's Therapeutic Research Institute (ATRI), Keck School of Medicine, University of Southern California, Los Angeles, CA, United States

**Keywords:** moyamoya disease, down syndrome, autoimmune disease, pediatrics, stroke

## Abstract

**Objective:** To determine if elevated rates of autoimmune disease are present in children with both Down syndrome and moyamoya disease given the high rates of autoimmune disease reported in both conditions and unknown etiology of angiopathy in this population.

**Methods:** A multi-center retrospective case-control study of children with Down syndrome and moyamoya syndrome, idiopathic moyamoya disease, and Down syndrome without cerebrovascular disease was performed. Outcome measures included presence of autoimmune disease, presence of autoantibodies and angiopathy severity data. Comparisons across groups was performed using the Kruskal-Wallis, χ2 and multivariate Poisson regression.

**Results:** The prevalence of autoimmune disease were 57.7, 20.3, and 35.3% in persons with Down syndrome and moyamoya syndrome, idiopathic moyamoya disease, and Down syndrome only groups, respectively (*p* < 0.001). The prevalence of autoimmune disease among children with Down syndrome and moyamoya syndrome is 3.2 times (*p* < 0.001, 95% CI: 1.82–5.58) higher than the idiopathic moyamoya group and 1.5 times (*p* = 0.002, 95% CI: 1.17–1.99) higher than the Down syndrome only group when adjusting for age and sex. The most common autoimmune diseases were thyroid disorders, type I diabetes and Celiac disease. No individuals with idiopathic moyamoya disease had more than one type of autoimmune disorder while 15.4% of individuals with Down syndrome and moyamoya syndrome and 4.8% of individuals with Down syndrome only had >1 disorder (*p* = 0.05, 95%CI: 1.08–6.08).

**Interpretation:** This study reports elevated rates of autoimmune disease in persons with Down syndrome and moyamoya syndrome providing a nidus for study of the role of autoimmunity in angiopathy in this population.

## Introduction

Moyamoya disease (MMD) is a chronic, progressive, cerebrovascular disorder and a leading cause of stroke in children and young adults. Classically, bilateral stenosis of the proximal intracranial arteries develops over time, leading to the development of abnormal and friable collateral vessels which creates the characteristic “puff of smoke” appearance on cerebral angiography (1–3). While the etiology of MMD remains unknown, several studies have identified a variety of autoimmune diseases associated MMD (4–11). Further, a recent reports suggest that a more aggressive disease course in MMD is associated with the presence of thyroid autoantibodies (12–14).

There is a well-established association between Down syndrome (DS) and the development of moyamoya syndrome (MMS) (7, 15, 16) with particularly high rates in children (17). Higher rates of both autoimmune disease and thyroid autoantibodies in this population raise additional concerns that these factors may yield a greater risk of the development of MMS although this has never been studied (18–24).

We hypothesize that individuals with both DS and associated MMS (DS/MMS) have higher rates of autoimmune disease, and when present, may be associated with more severe clinical presentations. The objective of this study was to assess whether prevalence of autoimmune disease and presence of systemic autoantibodies to the thyroid and other organs are elevated in individuals with DS/MMS.

## Methods

### Data Availability

Qualified investigators may request access to anonymized data. All proposals will require IRB authorization.

### Patient Selection

Institutional Review Board (IRB) approval was obtained for this study at all sites with waiver of consent granted. Patients with DS and DS/MMS were identified retrospectively from prospectively enrolled research databases at each of the four institutions contributing data, all of which were tertiary academic medical centers. Patients with MMD and MMS without DS were retrospectively identified from existing clinical databases using ICD-9 and ICD-10 codes. Patients seen between January 1, 2008 to December 31, 2020 were included. Some individuals included in this study had other, unrelated, data previously published (17, 25).

### Inclusion

Inclusion and exclusion criteria varied by group. For individuals with DS, patients required a previous diagnosis of DS (confirmed by genetic testing) and at least one clinical visit at a participating institution. Individuals with MMD or DS/MMS required a radiographically confirmed diagnosis of moyamoya angiopathy (3) and an evaluation by a pediatric neurosurgeon confirming the diagnosis. Individuals with DS/MMS were required to meet inclusion criteria for both groups. Both patients with unilateral and bilateral MMD/MMS were included in this study. Exclusion criteria for all groups included (1) incomplete clinical data, (2) prior chemotherapy or radiotherapy to the body and/or brain, (3) another non-DS genetic disorder (including but not limited to Alagille syndrome, neurofibromatosis type 1, sickle cell disease, or William's syndrome), and (4) and age <2 years or >19 years. For patients in the DS/MMS and MMD groups, additional exclusion criteria included clinically significant, sub-optimally treated, or untreated thyroid disease or obstructive sleep apnea (OSA) as it was unclear if this may influence development of cerebrovascular disease in this popuation (Appendix 1). Definitions for these disease entities are also included in Appendix 1.

### Definition of Autoimmune Disease

Autoimmune disease was identified using ICD-9 and ICD-10 coding (Appendix 2). For autoimmune thyroid disease, patients were required to either have a diagnosis of Hashimoto's thyroiditis or Graves' disease and have previously documented abnormal thyroid antibody studies and/or thyroid autoantibodies. Individuals with abnormal thyroid autoantibodies and no autoimmune diagnosis were not considered to have autoimmune disease. Thyroid levels (TSH and free/total T4) were not verified, nor was therapeutic intervention. Autoimmune disease in first-degree relatives was obtained solely from clinical documentation review and required a biological parent with a documented autoimmune disorder. Due to heterogeneity in documentation of sibling medical information and number of siblings, this information was not collected for analysis. As family member records were not included as part of data review in this study, there was no ability to verify this data.

### Biomarkers of Autoimmunity

All records were reviewed for the presence of positive thyroid peroxidase (TPO), anti-thyroglobulin (Tg) and glutamic acid decarboxylase 65-kd isoform (GAD65) antibodies. The presence of these antibodies were subsequently used to evaluate for the presence of thyroid dysfunction (TPO and anti-Tg antibodies) or type one diabetes (GAD65) to evaluate associations between biomarkers of autoimmunity and presence of autoimmune disease. In addition, presence of these biomarkers were also used to evaluate if clinical features of MMS (e.g. Suzuki score) were augmented by the presence in the serum.

### MMS/MMD

Neurovascular imaging and reports were reviewed for all patients in the MMD and DS/MMS groups to confirm the diagnosis of MMD or MMS. The diagnosis of MMD or MMS was evaluated for each case using 2012 international guidelines. (26) Suzuki scores were applied retrospectively to all cases when not available in documentation per established grading criteria. (27) Additional neurovascular biomarkers of disease severity such as laterality of disease, involvement of the posterior circulation, presence of collaterals and hemorrhage were also collected. Post-operative outcomes were not followed in this study.

### Statistical Analysis

Descriptive statistics were calculated for the primary outcome (autoimmune disease) and exposure variables. Median and interquartile ranges were reported for variables measured on the continuous (e.g., age) and ordinal (e.g., Suzuki score) scales. Proportions and corresponding 95% confidence intervals were calculated. Comparisons across groups was performed using the Kruskal-Wallis test for continuous/ordinal variables, and χ^2^ test with odds ratio (OR) for categorical variables, respectively. In comparing groups with heterogenous biomarker testing (e.g. TPO antibody testing was variable in each group), comparison of positive and negative values were only compared in tested individuals (unknown results were excluded from analysis). The multivariate Poisson regression model with robust error estimates were fit to estimate prevalence ratios (with DS/MMS group as reference), adjusted for age, sex, ethnicity, and autoantibodies collected.

## Results

**Figure 1:**
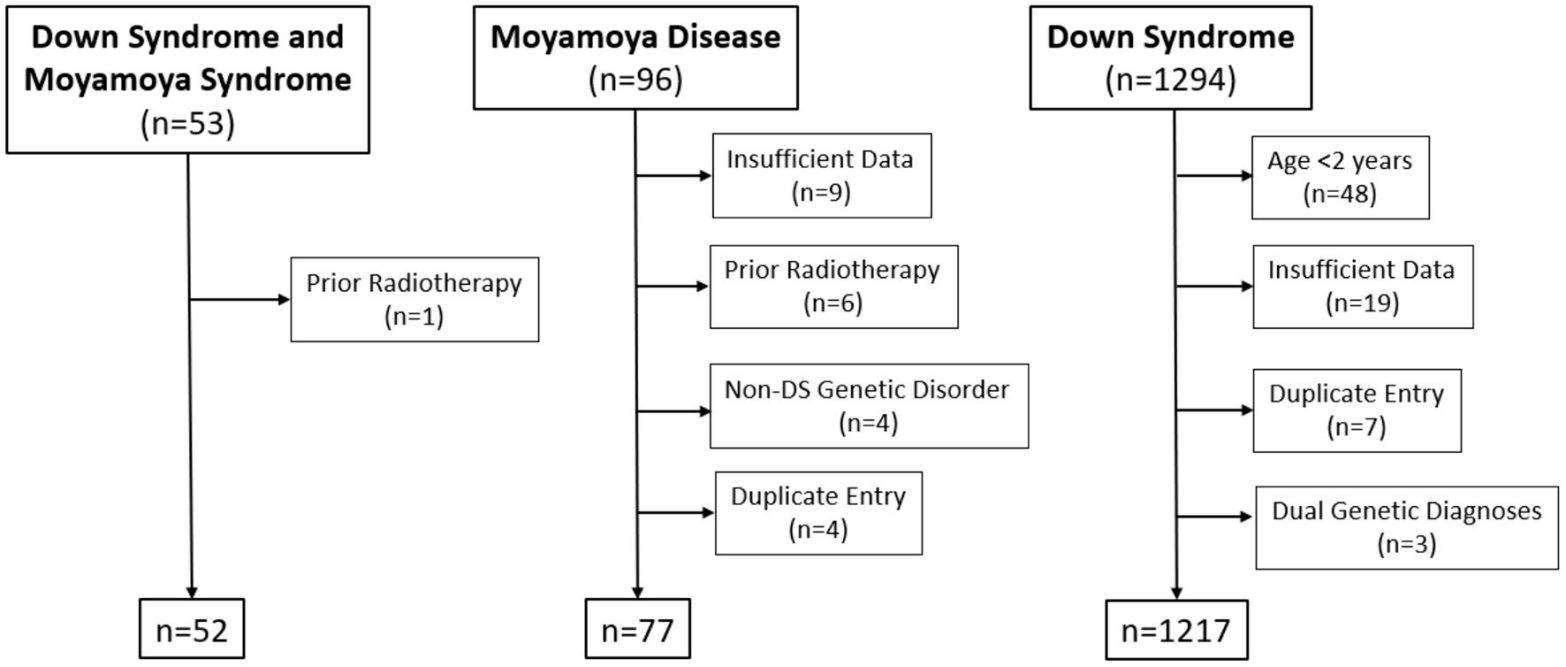
CONSORT diagram.

A total of 53 individuals with DS/MMS, 96 individuals with MMD, and 1,265 individuals with DS were initially identified, and 52, 77, and 1,217, respectively, met criteria for inclusion (Figure 1). The most frequent reasons for exclusion were age <2 years (*n* = 48), incomplete data (*n* = 27) and duplicate entries (*n* = 11). Two patients (one with MMD and one with DS/MMS) were excluded for clinically significant, untreated, OSA. All patients with MMD and DS/MMS had clinical neurovascular data and personal medical history available. Thyroid autoantibodies (TPO and anti-Tg) were available in 39, 45 and 83% of individuals with MMD, DS, and DS/MMS, respectively. Further, GAD 65 autoantibodies were available in 32, 61, and 56% of individuals with MMD, DS, and DS/MMS, respectively.

Clinical and demographic data are presented in Table 1. A clinical history of OSA was much more common (65.4 and 70.1%) in persons with DS/MMS and DS compared to MMD (4.6%) (*p* < 0.001, 95%CI: 6.7–375). The prevalence of autoimmune thyroid disease was 50.0% in persons with DS/MMS, which was significantly higher than both individuals with MMD (7.8%, *p* < 0.001, 95%CI: 3.74–27.62) and DS alone (25.7%, *p* = 0.01, 95%CI: 1.63–5.10).

**Table 1:** Demographics and clinical characteristics.

	**DS/MMS (*n* = 52)**	**MMD (*n* = 77)**	**DS (*n* = 1,217)**	***p*-value**	**95% CI**
**Sex (** ***n*** **, %)**					
Male Female	23 (44.2) 29 (55.8)	32 (41.6) 45 (58.4)	578 (47.5) 638 (52.5)	0.554	0.87–1.16
**Race/Ethnicity**					
Caucasian Hispanic Asian Black Other	85 (72,93) 37 (24,51) 10 (3,21) 4 (0, 13) 2 (0, 10)	73 (61, 82) 40 (29,52) 21 (12, 32) 5 (1, 13) 1 (0, 7)	87 (85,89) 35 (33,38) 9 (8,11) 4 (3,5) 0 (0, 1)	0.011	0.88–1.19
Age (median, IQR)	13 (8.25–17)	12 (6–15)	11 (7–15)	0.074	0.96–1.02
**History of Hypothyroidism (%, 95% CI)**					
Yes No Unknown	50 (36, 64) 50 (36, 64) 0	8 (3, 16) 79 (68, 88) 13 (6, 23)	15 (13, 17) 43 (40, 45) 43 (40, 46)	<0.001	
**Moyamoya Clinical Data**					
	**% (95% CI)**	**% (95% CI)**	***p*** **-value**		
**Clinical presentation**					
CVA TIA Headache Seizure Weakness Incidental	71 (57, 83) 8 (2, 19) 4 (0, 13) 10 (3, 21) 8 (2, 19) 0	68 (56, 78) 12 (5, 21) 10 (5, 19) 9 (4, 18) 0 1 (0, 7)	0.111		
**Preceding trigger** URI/Viral syndrome Trauma Other	23 (13, 37) 19 (10, 33) 0 4 (0, 13)	23 (14, 34) 22 (13, 33) 1 (0, 7) 1 (0, 7)	0.254		
Collaterals Present	79 (65, 89)	81 (70, 89)	0.826		
**Laterality**					
Bilateral Unilateral Left Right	73 (59, 84) 27 (16, 41) 17 (8, 30) 10 (3, 21)	79 (68, 88) 21 (12, 32) 13 (6, 23) 8 (3, 16)	0.775		
Suzuki grade (median, IQR)	3 (2.25–4)	3 (3–4)	0.360		
Hemorrhage (*n*, %)	0	3 (3.9)	0.273		
Aneurysm (*n*, %)	1 (1.9)	0	0.403		
Posterior circulation involvement (*n*, %)	14 (26.9)	11 (19.5)	0.391		

*CVA, cerebrovascular accident; IQR, interquartile range; OSA, obstructive sleep apnea; TIA, transient ischemic attack; URI, upper respiratory tract infection*.

**Figure 2:**
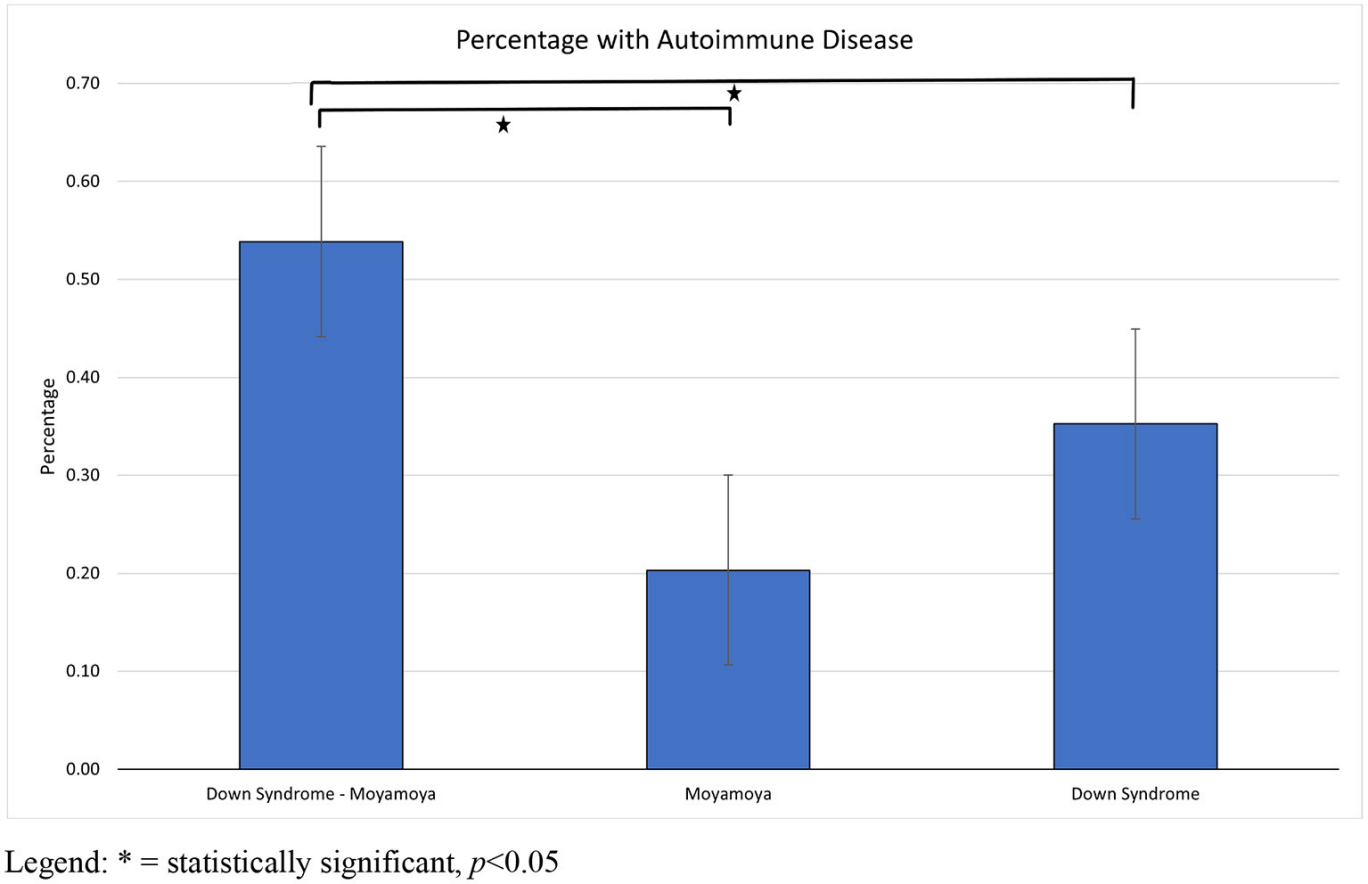
Rates of autoimmune disease by medical condition.

Rates of autoimmune disease between groups are presented in Table 2. The prevalence of personal autoimmune disease was 53.8% (95% CI: 39–68%) in DS/MMS, 35% (95% CI: 33–38%) in DS without MMS and 17% (95% CI: 9–27%) in MMD. This difference was strongly statistically significant difference between DS/MMS and DS and DS/MMS and MMD (*p* < 0.001). Poisson regression analysis revealed no impact from sex (*p* = 0.97, 95% CI: 0.87–1.16) or race/ethnicity (*p* = 0.73, 95% CI: 0.9–1.21) on autoimmune disease. The prevalence of autoimmune disease among children with DS/MMS is 3.2 times (*p* < 0.001, 95% CI: 1.82–5.58) higher than the MMD group and 1.5 times (*p* = 0.002, 95% CI: 1.17–1.99) higher than the DS group when adjusting for age, race/ethnicity, and sex (Figure 2). The presence of autoimmune thyroid disease significantly elevated in persons with DS/MMS compared to DS (*p* = 0.05, OR: 1.99, 95%CI: 1.06–3.74), and MMD (*p* = 0.007, OR: 4.36, 95%CI: 1.56–12.27). Individuals with DS/MMS were more likely to have more than one autoimmune disease compared to individuals with DS alone (*p* = 0.05, 95%CI: 1.08–6.08) with no individuals with MMD having more than one autoimmune disease. A family history of autoimmune disease more prevalent in persons with DS/MMS compared to MMD (*p* = 0.01, 95%CI: 1.33–5.99) and DS alone (*p* < 0.001, 95%CI: 6.94–22.5). After adjusting for the presence of TPO and anti-Tg antibodies, sex, ethnicity, and age, the prevalence ratio was 2.24 and 1.89 (*p* = 0.002, 95%CI: 1.35–3.68 and *p* = 0.02, 95%CI: 1.09–3.28, respectively). After adjusting for the presence of all autoantibodies (TPO, anti-Tg, and GAD65), sex, ethnicity, and age, the prevalence ratio was reduced slightly to 2.32 and 1.64 (*p* = 0.001, 95%CI: 1.41–3.84 and *p* = 0.08, 95%CI: 0.94–2.86, respectively).

**Table 2:** Personal and familial autoimmune disease.

	**DS/MMS (*n* = 52) % (95% CI)**	**MMD (*n* = 77)** **% (95% CI)**	**DS (*n* = 1217) % (95% CI)**	***p-*value**
**AUTOIMMUNE DISEASE**				
Any Thyroid Disorders TPO Ab positive (*n*, % of tested) Anti-Tg Ab positive (*n*, % of tested) Type I DBM GAD65 Ab positive (*n*, % of tested) Celiac Vitiligo JIA SLE Other	54 (39, 68) 27 (16, 41) 12, 92 11, 85 12 (4, 23) 6, 100 8 (2, 19) 0 (–, –) 6 (1, 16) 0 (–, –) 2 (0, 10)	17 (9, 27) 8 (3, 16) 1, 20 1, 20 4 (1, 11) 1, 100 3 (0, 9) 1 (0, 7) 0 (–, –) 0 (–, –) 1 (0, 7)	35 (33, 38) 16 (14, 18) 75, 39 75, 39 3 (2, 4) 17, 81 5 (4, 6) 1 (1, 2) 2 (1, 3) 0 (0, 1) 8 (7, 10)	<0.001 0.014 0.012 0.426 0.732 0.084 1.000 0.018
>1 Autoimmune disorder	15 (7, 28)	0 (–, –)	5 (4, 6)	<0.001
**1** ^**st**^ **DEGREE RELATIVE WITH AUTOIMMUNE DISEASE**				
Any Thyroid Disorder Type I DBM Celiac RA Vitiligo Alopecia Ulcerative Colitis Other	46 (32, 61) 6 (1, 16) 0 (–, –) 4 (0, 13) 8 (2, 19) 0 (–, –) 0 (–, –) 4 (0, 13) 25 (14, 39)	25 (16, 36) 12 (5, 21) 3 (0, 9) 3 (0, 9) 3 (0, 9) 1 (0, 7) 1 (0, 7) 0 (–, –) 3 (0, 9)	7 (5, 8) 5 (3, 6) 0 (0, 1) 0 (0, 1) 0 (0, 2) 1 (0, 1) 0 (0, 0) 0 (–, –) 0 (0, 1)	<0.001 0.027 0.106 0.004 0.001 0.261 0.183 0.001 <0.001
**TPO Antibodies**				
Positive Negative Not Tested^*a*^	52 (38, 66) 31 (19, 45) 17 (8, 30)	12 (5, 21) 32 (22, 44) 56 (44, 67)	9 (7, 11) 30 (27, 33) 61 (58, 64)	<0.001^a^
**Anti-Tg Antibodies**				
Positive Negative Not Tested^*a*^	46 (32, 61) 36 (24, 51) 17 (8, 30)	10 (5, 19) 34 (23, 45) 56 (44, 67)	9 (7, 11) 30 (27, 33) 61 (58, 64)	<0.001^a^
**GAD65 Antibodies**				
Positive Negative Not Tested^*a*^	25 (14, 39) 31 (19, 45) 44 (30, 59)	5 (1, 13) 26 (17, 37) 69 (57, 79)	2 (1, 3) 17 (15, 19) 81 (79, 83)	<0.001^a^

*DBM, diabetes mellitus; GAD, glutamic acid decarboxylase; JIA, juvenile idiopathic arthritis; SLE, systemic lupus erythematosus; TPO, thyroid peroxidase; Tg, thyroglobulin*.

a *Individuals who were not tested for autoantibodies were excluded from analysis, thus p values are a representation of positive and negative values in a limited cohort*.

Secondary analysis of individuals with and without autoimmune disease and clinical presentations is presented in Table 3. Neither sex nor race/ethnicity were statistically significant between groups or on sub-group analysis. TPO and anti-thyroglobulin antibodies were strongly associated with the presence of autoimmune disease in persons with DS/MMS (*p* < 0.001, 95%CI: 2.46–44.8) and DS (*p* < 0.001, 95%CI: 3.56–11.4) but not MMD (*p* = 0.44, 95%CI:0.4–35.4). GAD65 antibodies were not predictive of autoimmune disease in persons with DS/MMS (*p* = 0.119, 95%CI: 0.03–1.10), MMD (*p* = 0.99, 95%CI: 0.02–4.92) although were predictive of autoimmune disease in persons with DS (*p* < 0.001, 95%CI: 2.90–167).

**Table 3:** Clinical data for individuals with co-morbid autoimmune disorders.

	**DS/MMS AI (*n* = 28) % (95% CI)**	**DS/MMS Non-AI (*n* = 24)** **% (95% CI)**	**MMD AI (*n* = 13) % (95% CI)**	**MMD Non-AI (*n* = 64)** **% (95% CI)**	**DS AI (*n* = 428) % (95% CI)**	**DS Non-AI (*n* = 789)** **% (95% CI)**
Male sex	50 (30, 69)	38 (19, 59)	31 (9, 61)	44 (31, 57)	47 (43, 52)	48 (44, 54)
**Race/Ethnicity**						
Caucasian Hispanic Asian Black Other	89 (72, 98) 39 (22, 59) 7 (1, 24) 4 (0, 18) 0 (–, –)	79 (58, 93) 33 (16, 55) 13 (3, 32) 4 (0, 21) 4 (0, 21)	54 (25, 81) 54 (25, 81) 31 (9, 61) 15 (2, 45) 0 (–, –)	77 (64, 86) 38 (26, 50) 19 (10, 30) 3 (0, 11) 2 (0, 1)	88 (84, 91) 36 (31, 40) 9 (7, 13) 3 (2, 5) 0 (–, –)	86 (84, 89) 35 (32, 39) 9 (7, 12) 4 (3. 5) 1 (0, 2)
Age (median, IQR)	14 (7.5–17)	12 (8.25–17)	15 (13–17)	12.5 (6.5–17)	11 (7–15)	11 (7–15)
**TPO Antibodies**						
Positive Negative Not Tested	75 (55, 89) 14 (4, 33) 11 (2, 28)	25 (10, 47) 50 (29, 71) 25 (10, 47)	8 (0, 36) 62 (32, 86) 31 (9, 61)	13 (6, 23) 27 (16, 39) 61 (48, 73)	22 (18, 26) 42 (38, 47) 36 (31, 40)	2 (1, 3) 23 (20, 26) 75 (72, 78)
**Anti-Tg Antibodies**						
Positive Negative Not Tested	64 (44, 81) 25 (11, 45) 11 (2, 28)	25 (10, 47) 50 (29, 71) 25 (10, 47)	8 (0, 36) 62 (32, 86) 31 (9, 61)	11 (5, 21) 28 (18, 41) 61 (48, 73)	22 (18, 26) 42 (38, 47) 36 (31, 40)	2 (1, 3) 23 (20, 26) 75 (72, 78)
**GAD65 Antibodies**						
Positive Negative Not Tested	39 (22, 59) 29 (13, 49) 32 (16, 52)	8 (1, 27) 33 (16, 55) 58 (37, 78)	8 (0, 36) 16 (2, 45) 77 (46, 95)	5 (1, 13) 28 (18, 41) 67 (54, 78)	5 (3, 7) 23 (19, 27) 72 (68, 76)	0 (–, –) 14 (11, 16) 86 (83, 88)
**MOYAMOYA CLINICAL DATA**							
Presenting with CVA	71 (51, 87)	71 (49, 87)	46 (19, 75)	72 (59, 82)		
Collaterals Present	71 (51(87)	88 (68, 97)	77 (46, 95)	81 (70, 90)		
**Laterality**						
Bilateral Unilateral Left Right	68 (48, 84) 32 (16, 52) 21 (8, 41) 11 (2, 28)	79 (58, 93) 21 (7, 42) 13 (3, 32) 8 (3, 17)	77 (46, 95) 23 (5, 54) 15 (2, 45) 8 (0, 36)	80 (68, 89) 20 (11, 32) 13 (6, 23) 5 (38.5)		
Suzuki Grade (median, IQR)	3.0 (2–4)	3 (3–4)	3 (2–4)	3 (2.75–5)		
Hemorrhage	0 (–, –)	0 (–, –)	0 (–, –)	5 (1, 13)		
Aneurysm	4 (0, 18)	0 (–, –)	0 (–, –)	0 (–, –)		
Posterior Circulation Involvement TPO positive	18 (6, 37) 82 (63, 94)	38 (19, 59) 63 (41, 81)	15 (2, 45) 85 (55, 98)	20 (11, 32) 80 (68, 89)		

*AI, Autoimmune disease; CVA, cerebrovascular accident; GAD, glutamic acid decarboxylase; IQR, interquartile range; NS, non-significant; TPO, thyroid peroxidase; Tg, thyroglobulin*.

The presence of an autoimmune disorder did not influence disease severity in individuals with DS/MMS and MMD. Biomarkers of disease severity such as stroke at presentation (*p* = 0.61, *p* = 0.07), extent of collateral formation (*p* = 0.14, *p* = 0.49), bilateral disease (*p* = 0.73, *p* = 0.86), and posterior circulation involvement (*p* = 0.11, *p* = 0.51) were not significantly different between individuals with DS/MMS or MMD respectively. In individuals with unilateral angiopathy, there was no association between laterality and the presence of autoimmune disease (*p* = 0.78).

TPO positive individuals with DS/MMS or MMD did not have higher Suzuki scores (*p* = 0.74, *p* = 0.21), higher rates of bilateral disease (*p* = 0.98, *p* = 0.85) nor were more likely to have posterior circulation involvement (*p* = 0.49, *p* = 0.21), respectively. Similarly, the presence of anti-GAD65 antibodies did not correlate with Suzuki scores (*p* = 0.13, *p* = 0.43), bilateral disease (*p* = 0.71, *p* = 0.69) or posterior circulation involvement (*p* = 0.27, *p* = 0.84), although this biomarker was infrequently collected (*n* = 29 in DS/MMS and *n* = 15 in MMD).

## Discussion

Amongst individuals with DS/MMS, the prevalence of co-occurring autoimmune disease is elevated when compared to individuals with idiopathic MMD and DS alone. Although all groups demonstrated higher rates of autoimmune disease than neurotypical individuals, (20–24, 28) the high rates of both personal and parental autoimmune disease in persons with DS/MMS suggest a possible autoimmune or inflammatory component to the development of moyamoya angiopathy in individuals with DS.

In both the DS/MMS and MMD cohorts, the presence of autoimmune disease was not associated with disease severity or the likelihood of presentation with stroke. These findings are consistent with previous descriptions in adults with idiopathic MMD, wherein patients with autoimmune disease were more likely to have unilateral angiopathy at diagnosis, and thus, lower Suzuki scores (9). However, studies have reported that even though MMD may be diagnosed earlier in these populations, disease progression may in fact occur at a more rapid rate (29). This latter finding highlights the importance of early detection in individuals with autoimmune disease and further stresses the potential need for screening in asymptomatic individuals with DS, particularly in those with autoimmune disorders.

Autoimmune disease in individuals with idiopathic MMD has previously been reported in adult populations although these studies have been limited to homogenous Asian (9, 10) or Caucasian cohorts (30, 31). In our idiopathic MMD cohort, prevalence of autoimmune disease was roughly 17% which is similar to other reported rates in adults which range from 15–30% (9, 10, 30, 31). Literature on autoimmune disease in pediatric-onset MMD is much more sparse, restricted largely to smaller case series. However, it would be presumed that the rates of autoimmune disease would likely be lower in children given that they have fewer total years to develop such conditions and have a more limited number of exposures to potential triggers. For this reason, the very high rates of autoimmune disease observed in children with DS/MMS is even more striking.

The pathogenesis of moyamoya angiopathy remains unclear and both genetic and immune/inflammatory components to this disease have been hypothesized. While roughly 10% of cases of MMD are associated with genetic defects (notably chromosomes 3p24-26, 6q25, 8q23, 10q23.31, 12p12, and 17q25), none are associated with chromosome 21 which is the predominant cause of pathology in DS (32–37). In individuals with idiopathic MMD, growth factors such as vascular endothelial growth factor (VEGF), fibroblast growth factor, platelet-derived growth factor (PDGF) and multiple cytokines such as matrix metalloproteinases, hypoxia inducible factor 1-alpha, and cellular retinoic binding protein-1 have been reported to be elevated, although it remains unclear whether any of these biomarkers play a primary or secondary role in the vascular condition at hand (11, 38–41). It is unknown if similar expression patterns are observed in individuals with DS/MMS although the data presented in this report does indicate that inflammatory profiles may be present based on the high prevalence of co-morbid autoimmune disease in this population.

Interestingly, individuals with DS have been reported to have pro-inflammatory milieus even in the absence of cerebrovascular disease, (42–44) raising suspicion that pro-inflammatory states may predispose to the development of MMS in this population. While the systemic immune dysregulation observed in individuals with DS is presumed to be genetic, the possibility of a dual-genetic phenomenon, such as clustered HLA genotypes or dual genetic diagnoses, could be contributing to the proinflammatory state as well (45). Further study is needed to identify the connection between the overexpression of endothelial and systemic markers of inflammation and moyamoya angiopathy in patients with DS. Although there were elevated rates of TPO, anti-Tg and GAD65 antibodies in the DS/MMS group, the clinical significance of this was confounded by the heterogeneity of testing observed in this study. The adjusted prevalence scores which account for the presence or absence of the autoantibodies assessed in this study explain a portion of the difference in autoimmunity across groups. Thyroid autoantibodies accounted for approximately 40% variance in persons with DS/MMS and 8% variance in persons with DS alone although this increased to 48 and 11% respectively when adjusting for GAD65. This is a limitation of the data set although prevalence ratios were decreased without changes in statistical significance indicating a sustained effect on autoimmunity driving the presence of MMD. It is the authors' hypothesis that these autoantibodies are unlikely to be pathogenic in the development of angiopathy in the cases reported although are potentially representative of an inflammatory environment and predilection for autoimmunity.

This study is not without limitations. Although we report a large cohort of patients with rare disease, this study remains retrospective in design and reliant on appropriate documentation of medical record diagnoses. This resulted in heterogeneity in the data that was available for each patient, especially with regards to obtainment of biomarkers of autoimmunity although the large comparator group of individuals with only DS still allows for reasonable comparison. Similarly, bias may have influenced physician need to investigate for autoimmune disease in individuals with DS/MMS This may have skewed results in disease states where there were lower rates of testing such as MMD. Time of diagnosis for autoimmune disease was not available for most patients and thus it was not possible to determine the temporal relationship between the development of MMD/MMS and manifestations of autoimmune disease in our DS/MMS and MMD cohorts. Further, longitudinal assessment of disease progression could not be performed in this study due to its retrospective nature and limited available data. Although ICD-9 and ICD-10 diagnostic coding is generally considered to be accurate and reflective of disease pathology, an independent centralized diagnostic study review was not feasible in this study and diagnostic criteria utilization of may have fluctuated between centers and physicians. Report of autoimmunity in family members was very limited and must be interpreted with caution because of the retrospective, chart-based, methodology which limited the ability to confirm diagnoses. Because all four centers involved in this study are tertiary academic medical centers, severity bias may lead to higher rates of autoimmune disease than are present in the general population. Further, as three centers were located in the state of California, a regional skew towards populations of Hispanic and Asian descent, or particular environmental exposures, may limit generalizability of the findings to other populations within North America.

## Conclusions

This study reveals elevated rates of autoimmune disease in children with DS/MMS, when compared to MMD and DS alone. Although individuals with DS have a known proclivity for endothelial and systemic inflammation, as well as autoimmune disease in general, these data indicate that autoimmunity could potentially play a unique role in the pathogenesis of MMS in persons with DS. Further investigation into the potential role of inflammation as an etiology or inciting factor in the development of MMS in patients with DS is warranted.

## Data Availability Statement

The original contributions presented in the study are included in the article/Supplementary Material, further inquiries can be directed to the corresponding author/s.

## Ethics Statement

The studies involving human participants were reviewed and approved by Children's Hospital Los Angeles/Keck School of Medicine IRB. Written informed consent to participate in this study was provided by the participants' legal guardian/next of kin.

## Author Contributions

JS and SL were responsible for drafting/revision of the manuscript for content, including medical writing for content. They had a major role in the acquisition of data. Responsible for study concept, design, analysis and interpretation of data. AW responsible for acquisition of the data, analysis, and interpretation of the data. EH was responsible for the aquision of the data, study concept and design. She also performed analysis and interpretation of the data. DN played a major role in acquision of data. She assisted with study concept and design. MK and RT were responsible for acquisition of the data and drafting of the manuscript. RD-A performed primary and secondary analysis and interpretation of the data and all statistical analysis. MM played a major role in the acquisition of the data and assisted with the interpretation of data. She also revised and edited the manuscript for intellectual content. BS was responsible for drafting and revising the manuscript for intellectual conent and supervised data collection. He also assisted with analysis and interpretation of the data. GS was responsible for study concept and design. He assisted with analysis and interpretation of the data and assisted with revision and editing of the manuscript for intellectual content. MR was responsible for drafting/revision of the manuscript for content, including medical writing for content. He had a major role in the acquisition of data and supervised data collection. He was responsible for study concept and design and analysis and interpretation of data. All authors contributed to the article and approved the submitted version.

## Conflict of Interest

The authors declare that the research was conducted in the absence of any commercial or financial relationships that could be construed as a potential conflict of interest.

## Publisher's Note

All claims expressed in this article are solely those of the authors and do not necessarily represent those of their affiliated organizations, or those of the publisher, the editors and the reviewers. Any product that may be evaluated in this article, or claim that may be made by its manufacturer, is not guaranteed or endorsed by the publisher.

## Abbreviations

CI: confidence interval
CVA: cerebrovascular accident
DS: Down syndrome
GAD65: glutamic acid decarboxylase 65-kd isoform
IRB: internal review board
ICD: international classification of diseases
MMD: moyamoya disease
MMS: moyamoya syndrome
OR: odds ratio
OSA: obstructive sleep apnea
PDGF: platelet-derived growth factor
TIA: transient ischemic attack
TPO: thyroid peroxidase
Tg: thyroglobulin
URI: upper respiratory tract infection
VEGF: vascular endothelial growth factor.
