# Supplementary material for: Increased Autoimmunity in Individuals With Down Syndrome and Moyamoya Disease
Source: Front Neurol. 2021 Sep 8;12:724969. doi: 10.3389/fneur.2021.724969 (PMC8455812; doi:10.3389/fneur.2021.724969)
Supplement: Supplementary file 1 [file Data_Sheet_1.DOCX]

**Appendix 1:** definitions for “sub-optimally treated”, “untreated”, “clinically significant disease”, and “clinically insignificant disease” for patients with prior diagnosis of hypothyroidism or obstructive sleep apnea.

1. Hypothyroidism (defined either by prior ICD-9 or ICD-10 code, treatment with levothyroxine, or serum TSH >4.5)
   1. Sub-optimal treatment: defined as 2 or more changes to any medication in the past 2 years used to treat hypothyroidism or the use of a second (or more) agent for control of hypothyroidism.
   2. Untreated: defined as patients with a prior diagnosis of hypothyroidism who have not received any pharmacotherapy for less than or equal to one year.
   3. Clinically significant disease: defined as patients experiencing any clinical comorbidity that could be linked to hypothyroidism following laboratory diagnosis of hypothyroidism including but not limited to: fatigue, memory issues (excluding developmental delay given patient population), excessive weight gain, chronic constipation, active or history of pleural effusions, hair loss, myxedema, bradycardia (persistent), menstrual irregularities, or thyroid crisis. All patients with history of auto-immune thyroid disease were excluded.
   4. Clinically insignificant disease: defined as any patient with a prior diagnosis of hypothyroidism as above treated on either no pharmacotherapy or monotherapy with either normalization of lab values or lack of any comorbid symptoms as defined above. No more than one alteration to drug monotherapy was permitted in the past year in this group.
2. Obstructive Sleep Apnea (defined as prior ICD-9 or ICD-10 diagnosis, sleep study with Apnea-hypopnea index (AHI) > 1.0, or clinical history of snoring with witnessed apnea)
   1. Sub-optimal treatment: defined as any patient requiring positive pressure ventilation with subsequent normalization of AHI to less than 5.0 but with more than 2 adjustments of positive pressure ventilation settings in the past 2 years.
   2. Untreated disease: diagnosis of obstructive sleep apnea as above but without any clinical intervention made
   3. Clinically significant disease: defined as any patient receiving positive pressure ventilation without correction of AHI to <5.0, or, patients with a diagnosis of obstructive sleep apnea and any co-morbid symptomatic condition including but not limited to: excessive daytime sleepiness, fatigue, need for daytime napping, irritability, attention issues (including diagnosis of ADHD), impulsivity or aggression at school (for patients <18 years), headache, chronic hypertension, or memory issues.
   4. Clinically insignificant disease: defined as patients with prior diagnosis of obstructive sleep apnea as above with normalization of AHI to less than 5.0 and with 1 or less adjustment of positive pressure ventilation in the past 2 years. Patients should have no co-morbid symptomatic conditions as defined above.
