# Supplementary material for: Increased Autoimmunity in Individuals With Down Syndrome and Moyamoya Disease
Source: Front Neurol. 2021 Sep 8;12:724969. doi: 10.3389/fneur.2021.724969 (PMC8455812; doi:10.3389/fneur.2021.724969)
Supplement: Supplementary file 2 [file Data_Sheet_2.DOCX]

**Appendix 2:** Autoimmune Conditions

| **Disease** | **ICD-9** | **ICD-10** |
| --- | --- | --- |
| *Cardiac* | | |
| Myocarditis | 391.2 or 429.0 | I40, I09 or I51.4 |
| *Dermatologic* | | |
| Alopecia areata | 704.01 | L63 |
| Autoimmune angioedema | 277.6 or 995.1 | D84.1 |
| Autoimmune urticaria | 708.0 | L50.8 |
| Bullous pemphigoid | 694.5 | L12 |
| Discoid lupus erythematous | 695.4 | H01.12 or L93 |
| Epidermolyisis bullossa | 694.8 | Q81 |
| Pemphigus vulgaris | 694.4 | L10 |
| Psoriasis | 696.0 | L40 |
| Systemic scleroderma | 710.1 | M34 |
| Vitiligo | 709.01 | L80 |
| *Endocrine* | | |
| Autoimmune pancreatitis | 577.1 | K86.1 |
| Diabetes mellitus type 1 | 250.01 | E10 |
| Autoimmune thyroiditis (Hashimoto’s) | 245.8 | E06.3 |
| Grave’s disease | 242.0 | E06.3 or Z86.39 |
| Sjogren syndrome | 710.2 | M35 |
| *Gastrointestinal* | | |
| Autoimmune hepatitis | 571.42 | K75.4 |
| Celiac disease | 579.0 | K90 |
| Crohn’s disease | 555.0 | K50 |
| Primary biliary cholangitis | 571.6 | K74.3 |
| Primary sclerosing cholangitis | 576.1 | K74.3 |
| Ulcerative colitis | 556.0 | K51 |
| *Hematologic* | | |
| Antiphospholipid antibody syndrome | 289.81 | D68.81 or D68.82 |
| Autoimmune hemolytic anemia | 283.0 | D59.1 or D59.13 |
| Autoimmune lymphoproliferative disorder | 279.41 | D89.82 |
| Autoimmune thrombocytopenic purpura | 287.31 | D69.3 |
| Evan’s syndrome | 287.32 | D69.41 |
| Pernicious anemia | 281.0 | D51.1 |
| *Multi-Organ* | | |
| Still’s disease | 714.2 | M06.1 |
| Ankylosing spondylitis | 720.0 | M45 |
| CREST syndrome | 710.1 | M34.1 |
| IgG-4 related disease | - | D89.89 |
| Juvenile idiopathic arthritis | 714.3 | M08 |
| Psoriatic arthritis | 696.0 | L40.5 |
| Rheumatoid arthritis | 714.0 | M05 |
| Sarcoidosis | 135.0 | D86 |
| Systemic lupus erythematosus | 695.4 | L93 |
| *Muscular* | | |
| Dermatomyositis | 710.3 | M33 |
| Myositis | 729.1 | M60.8 or M60.9 |
| Myasethenia gravis | 358.0 | G70 |
| Neuromyotonia/Isaac’s syndrome | 333.9 | G13 |
| *Nephrologic* | | |
| Anti-glomerlular basement membrane nephritis | 446.21 | N05.8 |
| Lupus nephritis | 583.81 | M32.14 |
| *Neurologic* | | |
| Acute disseminated encephalomyelitis | 323.61 or 323.81 | G04 and G04.81 |
| Acute motor axonal neuropathy | 356.8 | G61 and G61.8 |
| Anti-N-methyl-D-aspartate receptor encephalitis | - | G04.81 |
| Chronic inflammatory demyelinating polyneuropathy | 357.81 | G61.81 |
| Guillain-Barre syndrome | 357.0 | G61 |
| Hashimoto’s encephalopathy | - | G39.4 |
| Lambert-Eaton mysethenic syndrome | 358.1 | G70.8 |
| Multiple sclerosis | 340.0 | G35 |
| Stiff-person syndrome | 333.91 | G25.82 |
| Sydenham’s chorea | 392.0 | I02 or G25.5 |
| Transverse myelitis | 323.82 and 341.2 | G37.3 |
| *Ophthalmologic* | | |
| Autoimmune uveitis | 364.0 | H44.13 |
| Cogan syndrome | 370.52 | - |
| Neuromyelitis optica spectrum disorder | 341.0 | G36 |
| Opsoclonus-myoclonus ataxia syndrome | 379.59 | G25.3 |
| Optic neuritis | 377.3 | H46 |
| Scleritis | 379.0 | H15 |
| Susac syndrome | 348.39 | - |
| Tolosa-Hunt syndrome | 378.55 | H49.4 |
| *Vascular* | | |
| Behcet’s disease | 136.1 | M35.2 |
| Eosinophilic granumlomatosis with polyangiitis | 446.4 | M31.3 |
| Giant cell arteritis | 446.5 | M31.5 |
| Granulomatosis with polyangiitis | 446.4 | M31.3 |
| Kawasaki disease | 446.1 | M30.3 |
| Lupus vasculitis | 583.81 | L95 |
| Rheumatoid vasculitis | 447.6 | L95 |
| Polyarteritis nodosa | 446.0 | M30 |
| Polymalgia rheumatica | 725.0 | M35.3 |
| Primary or secondary vasculitis | 447.6 | M05.2, M31.8 or I77.6 |
